# Cancer du sein au Maroc: profil phénotypique des tumeurs

**DOI:** 10.11604/pamj.2016.25.74.9966

**Published:** 2016-10-06

**Authors:** Ahmadaye Ibrahim Khalil, Karima Bendahhou, Houriya Mestaghanmi, Rachid Saile, Abdellatif Benider

**Affiliations:** 1 Laboratoire de Physiopathologie et Génétique Moléculaire, Faculté des Sciences Ben M’Sik, Université Hassan II, Casablanca, Maroc; 2 Registre des Cancers de la Région du Grand Casablanca, Maroc; 3 Laboratoire de Biologie et Santé, Unité de Recherche Associée au CNRST-URAC 34, Faculté des Sciences Ben M’Sik, Université Hassan II, Casablanca, Maroc; 4 Centre Mohammed VI pour le traitement des cancers, CHU Ibn Rochd Casablanca, Maroc

**Keywords:** Cancer du sein, phénotype, pronostic, immunohistochimie, Breast cancer, phenotype, prognosis, immunohistochemistry

## Abstract

Le cancer du sein est le plus fréquent chez la femme et figure parmi les principales causes de mortalité liées au cancer. La curabilité de ce type tumoral est en augmentation, grâce aux programmes de dépistage et aux progrès thérapeutiques, qui ont certes augmenté la survie des patients. Mais des défis restent à relever en rapport avec l’instabilité phénotypique des cellules cancéreuses. L’objectif de ce travail est d’étudier le profil phénotypique du cancer du sein chez les patients pris en charge au Centre Mohammed VI pour le traitement des Cancers, durant les années 2013-2014. Il s’agit d’une étude transversale sur deux années, incluant les cas du cancer du sein pris en charge au Centre. Le recueil des données était fait à partir des dossiers des patients et analysés par le logiciel Epi Info. 1277 patients ont été pris en charge au sein de notre centre. 99,5% des cas de sexe féminin, l’âge moyen était 50,20 ± 11,34 ans. Le type histologique le plus fréquent était le carcinome canalaire infiltrant (80,7% des cas). Le stade diagnostic était précoce (56,9%). Le phénotype moléculaire le plus fréquent était le luminal A (41,4% des cas). Le luminal B, le HER2 et les triples négatifs étaient dans respectivement 10,4%, 6,3%, 11,2% des cas. L’étude du phénotype tumoral des patients atteints du cancer du sein permet l’orientation du clinicien dans le choix du traitement, et des décideurs dans la planification de programmes de lutte contre cette pathologie.

## Abstract (en)

Breast cancer is most common in women and it is among the leading causes of cancer related deaths. The curability of this type of tumor is increasing thanks to screening programs and treatment advances which have certainly enhanced patient survival. But challenges remain, particularly in respect of phenotypic instability of cancer cells. The aim of this study was to analyse the phenotypic profile of breast cancer in patients treated at Mohammed VI Cancer Treatment Center over the years 2013-2014. We conducted a cross-sectional study over a two-year period, including the cases of breast cancer treated in our Center. Data were collected from patients medical records and analyzed using Epi Info software. 1277 patients were treated in our Center. 99.5% were females, mean age 50.20 ± 11.34 years. The most common histological type was invasive ductal carcinoma (80.7% of cases). It was diagnosed at an early stage (56,9%). The most common molecular phenotype was luminal A (41.4% of cases). Luminal B, HER2 and triple negatives occurred in 10.4%, 6.3%, 11.2% of cases respectively. The study of tumor phenotype in patients with breast cancer helps clinician make treatment choice and policy makers implement programs against this disease.

## Introduction

Le cancer du sein est le cancer le plus fréquent chez la femme et représente la première cause de mortalité. C’est une pathologie éminemment hétérogène. De multiples classifications (cliniques, histopathologies ou moléculaires) ont vu le jour, afin de permettre l’identification précise des tumeurs les plus agressives et l’adaptation thérapeutique qui en découle. L’une des classifications les plus simples est basée sur les propriétés intrinsèques tumorales du cancer du sein, qui peuvent être classées en deux grandes catégories: les tumeurs qui expriment le récepteur aux œstrogènes (RE) (appelé tumeurs luminales ou RE+) et celles qui ne l’expriment pas ou RE- [1]. L’existence d’un système de classification histologique dans le cancer du sein est loin d’être précise pour prédire le pronostic d’un patient [2]. Morphologiquement, des tumeurs identiques peuvent présenter des résultats cliniques différents. Pour cela, une classification moléculaire permet principalement de déterminer différentes classes moléculaires appartenant à un type histologique similaire. Une bonne connaissance de ces classes moléculaires et de leur identification pourrait conduire à un diagnostic plus avancé et à un système pronostic plus bénéfique et précis [3]. Il est donc très important de connaitre le profil biologique du cancer du sein [4]. Le développement de méthodes moléculaire analytiques remonte à une vingtaine d’année, quant à l’immunohistochimie (IHC), c’est la première technique qui a permis de classer les tumeurs du sein. L’émergence d’hybridation in situ d’acide nucléique a conduit à l’identification de deux nouveaux groupes selon la surexpression du Her2 ou non [5]. Perou et al. étaient les premiers à établir le système de classification des tumeurs mammaires, basé sur l’analyse de l’expression des gènes et ont montré l’existence de quatre classes moléculaires du cancer du sein : le luminal-like, le basale-like, le normal-like et le HER2 enrichie [6]. D’autres études plus tard ont élargi le spectre d’analyse et ont montré l’existence de cinq classes moléculaires dans le cancer du sein: le luminal A, luminal B, Her2 enrichie, basale like et le normale like [7]. Des progrès importants ont été réalisés en immunohistochimie pour la mise en évidence de plusieurs marqueurs tumoraux d’intérêt pronostic et prédictif au profit de l’analyse de l’expression génique. Certains auteurs ont montré aussi qu’elle peut identifier de manière fiable les principales classes moléculaires du cancer du sein invasif [8]. L’intérêt de l’utilisation de nouveaux marqueurs immunohistochimiques tel que les cytokératines et le EGFR2 a été confirmé par plusieurs travaux [9, 10]. L’objectif du travail est d’étudier le profil phénotypique du cancer du sein chez les patients pris en charge au Centre Mohammed VI pour le traitement des Cancer, durant les années 2013-2014.

## Méthodes

**Type d’étude:** Il s’agit d’une étude transversale menée au sein du Centre Mohammed VI pour le traitement des cancers au Centre Hospitalier IBN ROCHD (l’un des deux grands centres pour la prise en charge et le traitement des cancers au Maroc).

**Population d’étude:** Nous avons inclus dans notre étude de manière consécutive tous les cas du cancer du sein ayant été pris en charge au centre du 01-janvier-2013 jusqu’au 31-décembre-2014. Le recueil des données a été fait de manière rétrospective à partir des dossiers médicaux des patients. Les données recueillis portaient sur: Données sociodémographiques: le sexe, l’âge au moment de diagnostic, l’âge de la puberté, le statut marital, l’âge à la première grossesse, le nombre d’enfant et la ménopause; Données cliniques: le stade au moment du diagnostic, la taille tumorale, l’envahissement ganglionnaire, la métastase à distance, le type histologique, le grade histologique, la latéralité avec le quadrant ou région tumorale, le statut des récepteurs hormonaux et le récepteur Her2 (Human Epidermal Growt factor) et l’indice de prolifération Ki-67; Données thérapeutiques: chimiothérapie néo-adjuvant, radiothérapie, chimiothérapie adjuvante, hormonothérapie et thérapie ciblées.

Le profil immunohistochimique des tumeurs était effectué sur la base des résultats anatomo-biologiques, basés sur l’évaluation des récepteurs d’œstrogènes et de progestérone, la surexpression de HER2 et l’index de prolifération Ki 67. Le marquage de ces récepteurs est considéré positif quand plus de 10% des cellules sont marquées, le récepteur HER2 a un score de 3+ et l’index de prolifération quand plus de 14% des cellules sont marquées. Selon les différents phénotypes obtenus, cinq sous-types phénotypes immunohistochimiques ont été définis: luminal A (ER+ et/ou PR+, HER2- et Ki-67< 14); luminal B (ER+ et / ou PR+, HER2- et Ki-67>14 ou ER+ et / ou PR+, HER2+); HER2 Positive (ER-, PR-, HER2+); le triple négatif ou basale like (ER-, PR-, HER2-); le non classé (absence de la surexpression de l’un de trois molécules).

Le stade au moment du diagnostic a été considéré précoce par le stade I et II et tardif par le stade III et IV. La saisie des données a été réalisée par Microsoft office Excel (2007) et l’analyse des variables par le logiciel Epi Info. L’étude association en croisant les variables entre les différents groupes a été évaluée par le test de chi-deux. Le test est considérée comme significatif lorsque p<0,05.

## Résultats

Un total de 1277 patients diagnostiqués pour un cancer du sein pris en charge au Centre Mohamed VI pour le traitement des cancers, la quasi-totalité était de sexe féminin (99,5% des cas), l’âge moyen des patients était de 50,20 ±11,34 ans, avec des extrêmes (17 - 93 ans). Le type histologique le plus fréquent était le carcinome canalaire infiltrant (80,70% des cas), viennent ensuite le carcinome invasif (8,1% des cas), le carcinome lobulaire et les autres types histologiques étaient respectivement 4,6% et 6,6% des cas. Le grade SBR le plus fréquent était le grade II (54,0% des cas), suivis du grade III (31,9% des cas), et le grade I qui était observé dans 3,9% des cas. L’étude clinico-pathologique a montré que le diagnostic du cancer du sein a été précoce dans 56,9% des cas, versus 43,1% qui étaient tardifs. 51,6% des patients ont eu un envahissement ganglionnaire et 7,7% ont développé des métastases à distance (Tableau 1). Pour le profil immunohistochimique, l’analyse a révélé que 41,4% étaient de type luminal A, 10,4% de type luminal B, 6,3% de type HER2 positive, 11,4% de type basale like ou triple négative et 30,4% sont non classé (absence d’information pour le biomarqueur étudié). La différence d’âge était significative chez les patients dans les différents groupes (Tableau 2). L’étude de croisement des différents groupes moléculaires avec le type histologique et le grade histologique SBR, le type histologique carcinome canalaire infiltrant était observé dans 44,4% des cas dans le luminal A, 13,2% des cas dans le luminal B, 7,7% des cas dans le HER2 positif et 12,8% des cas dans le triple négatif. Le luminal A est caractérisé par un bas grade (60,0% de grade I), le luminal B par un grade moyen (14,3% de grade II), le type HER2 positif et le triple négatif sont caractérisés par un grade histologique élevé, respectivement 8,5% et 17,9% de grade III (Tableau 3). Le stade de la tumeur lors du diagnostic était variable dans les différents groupes moléculaires, le stade II se localise dans le luminal A et le stade III dans les sous-groupes luminal B et Her2 positive (Tableau 3). Le luminal A présente un envahissement ganglionnaire et la métastase à distance. La plupart des patients ont subi un traitement chirurgical (80,5%). Selon le profil moléculaire établit, un pourcentage élevé de la chirurgie était dans le luminal A (45, 3%) et le luminal B (13,6%), le Her2 positive (7,2%) et le triple négatif (13,0%).

**Tableau 1:** Description générale de la population étudiée

Caractéristiques sociodémographiques	Pourcentage (%)	Effectif
**Age moyen (ans)**	50,20	1277
**Age moyen de la puberté (ans)**	13,72	1277
**Age moyen du premier enfant (ans)**	23,67	311
**Age moyen de la ménopause (ans)**	49,13	415
**Sexe**		
Masculin	0,5	6
Féminin	99,5	1271
**Statut marital**		
Célibataire	15,8	175
Marié	60,5	669
Divorcé	14,0	155
Veuf	9,6	106
**Contraceptif oral**		
Oui	71,4	242
Non	28,0	97
**Ménopause**		
Oui	32,2	415
Non	67,5	862
**Caractéristiques cliniques**		
I	3,9	50
II	54,0	690
III	31,9	407
**Taille tumorale**		
<2cm	76,8	981
>2cm	23,2	296
**Statut ganglionnaire N**		
N0	50,6	646
N+	49,4	631
**Statut métastatique M**		
M0	92,8	1185
M+	7,2	92
**Phénotype moléculaire**		
Luminal A	41,4	529
Luminal B	10,4	133
Her2 positive	6,3	81
Triple négatif	11,4	146
Non classés	30,4	388

**Tableau 2:** Caractéristiques épidémiologiques des patients selon le phénotype moléculaire

	Luminal A	Luminal B	Her2 positive	Basale like	Non classé	P value
**Age moyen (ans)**	50,7(±10,9)	47,5(±11,5)	49,7(±9,1)	47,9(±11,7)	51,2(±11,8)	0,01
**Antécédent familial du cancer du sein (%)**						
Oui	44,2	9,9	3,5	17,4	25,0	0,02
Non	41,0	10,5	6,8	10,5	31,2	
**Ménopause (%)**						
Oui	66,5	68,4	60,5	69,9	69,1	0,5
Non	33,0	31,5	39,5	30,1	30,1	

**Tableau 3:** Caractéristiques clinico-pathologiques des patientes

	Luminal A	Luminal B	Her2 positive	Triple négatif	Non classé	P value
**Type histologique**						
CCI	43,9	11,3	7,3	11,4	26,1	0,001
Autre	31,5	6,8	2,4	11,9	47,8	
**Grade SBR**						0,000
Grade I	6,3	9,6	3,7	6,7	43,7	
Grade II	47,4	11,9	5,9	9,0	25,7	
Grade III	32,9	8,6	8,1	17,0	33,4	
**Stade**						
Stade I et II	42,8	7,6	6,1	12,8	30,8	0,002
Stade III et IV	39,6	14,2	6,7	9,6	29,8	
**Statut ganglionnaire**						
N0	40,4	6,8	6,0	11,5	35,3	0,000
N1	42,5	14,1	6,7	11,4	25,4	
**Statut Métastasique**						
M0	41,4	10,1	6,7	12,1	29,7	0,005
M1	41,3	14,1	2,2	3,3	39,1	

## Discussion

Le cancer du sein est une maladie complexe et hétérogène, associée à des facteurs cliniques, pathologiques et biologiques variables d´une population à une autre. L’étude des profils d’expression génomique a apporté une classification moléculaire de la maladie en sous-groupes cliniquement pertinents. Cette classification a permis d’identifier plusieurs catégories de tumeurs mammaires, par l’expression différentielle des cDNA. La distinction majeure se fait d’abord selon le transcriptome des tumeurs ER positives et négatives. Les études du développement canalaire du sein normal suggèrent l’existence d’une cellule pro-génitrice qui selon deux lignées différentes, donne naissance à deux types des cellules matures: la cellule épithéliale luminale interne (glandulaire) et la cellule myoépithéliale externe [11]. Les protéines de filaments intermédiaires nommées cytokératines sont exprimées en combinaison distinctes dans ces deux différents types de cellules épithéliales. Les cytokératines CK8, CK18 et CK19 sont exprimés dans les cellules luminales. Par contre, les cytokératines basales tels que CK5, CK6 et CK14 sont exprimées dans la majorité des cellules myoépithéliales mammaires. Les études immunohistochimiques ont montré que l’expression des cytokératines est conservée pendant la cancérogenèse, ce qui permet la détermination des différents types phénotypiques des tumeurs mammaires: basale/myoépithélial et luminal. Selon les travaux basés sur le profil d’expression génomique, plusieurs auteurs ont proposés d’identifier les types moléculaires suivants: le type basale-like caractérisé par ER- (récepteur œstrogène négatif), RP- (récepteur progestérone négatif) et le Her2 négatif, le raccourci triple négatif est souvent utilisé pour qualifier ce type. Le type HER2 a été défini comme des tumeurs RH négatif et HER2 positif. Les tumeurs luminal-like étaient toutes des tumeurs RH positif. Plusieurs études ont confirmé les corrélations entre ces types moléculaires et les caractéristiques cliniques et histologiques des cancers mammaires in situ, des cancers invasifs, ainsi que du cancer du sein inflammatoire [12, 13]. Cette étude menée au sein du Centre Mohamed VI pour le traitement des cancers dont l’objectif était d’étudier le profil phénotypique du cancer du sein a révélé que le type le plus fréquent était le luminal A (41,4%) suivis des triple négatives ou basale like (11,4%), ensuite, viennent le luminal B (10,4%), le Her2 positive (6,3%) et (30,4%) des cas étaient non classés. Le phénotype luminal A était caractérisé par une forte expression des récepteurs aux œstrogènes et à la progestérone, alors que le Her2 est négatif, il exprime les cytokératines 8,18 et 19 et le GATA3 [12, 14]. Les phénotypes luminal A sont associés à des évolutions cliniques et des réponses thérapeutiques différentes et un bon pronostic. Dans notre série, le luminal A était le plus fréquent (41,1% des cas) avec un âge moyen au moment du diagnostic de 50,7 ans. Dans ce groupe, le type histologique le plus fréquent était le carcinome canalaire infiltrant, caractérisé par un bas grade histologique, grade II SBR et un indice de prolifération faible. Ces résultats concordent avec les données de la littérature [12, 15]. Carey LA et al. ont montré que la prévalence du phénotype luminal A varie de 54 à 74% des cas (p<0,001) [9]. Plusieurs études qui ont été réalisées sur des populations occidentales ont montrées que les luminal A étaient souvent les plus rependues et qui ont relativement un bon pronostic [15, 16]. Les tumeurs luminal A sont des tumeurs hormonosensibles et bénéficient d’un traitement hormonal [11, 14, 16].

Le phénotype luminal B possède un profil immunohistochimique positif aux récepteurs aux œstrogènes mais, sont moins exprimés dans le cas du luminal A, en plus d’une surexpression de Her2 et une forte expression de l’indice de prolifération ki-67 [17, 18], ce qui entraine un risque relatif de rechute (IC 95%), par rapport aux tumeurs luminales de faible prolifération [1, 7]. Les tumeurs des phénotypes luminal B présentent un comportement plus agressif [17–19]. Bien que les tumeurs luminal présentent une faible réponse à la chimiothérapie, la réponse clinique et pathologique à la chimiothérapie semble être plus élevée que celle des tumeurs luminal B [17]. Dans notre série, le phénotype luminal B était représenté dans 12,7% des cas, le grade histologique le plus fréquent étaient le grade II dans 14,3% des cas, l’envahissement ganglionnaire dans 15,3% des cas et une métastase a été retrouvée dans 12,5% des cas. Al tamimi et al. ont montré dans une série de 231 cas que le phénotype luminal B représente 16,0% des cas [10], alors que Cheang et al. ont montré une prévalence élevée du luminal B dans 32% des cas [13]. Ce résultat peut être expliqué par l’indice de prolifération très bas qu’ils ont utilisé par rapport aux précédentes études. D’ailleurs, certains auteurs ont montré que bien que l’indice de prolifération permet de bien identifier le luminal B, l’expression des récepteurs hormonaux et le statut du Her2 seuls peuvent être utilisés pour identifier les tumeurs luminal B, cela peut entrainer une augmentation de l’expression du luminal B [20]. Le pourcentage de l’expression de l’indice de prolifération était un sujet de controverse pour plusieurs études. Certains auteurs ont utilisé un seuil de 10% [21, 22], d’autres jusqu´au seuil de 20% [23]. Dans notre étude, on a utilisé un seuil de marquage de 14%. Les tumeurs de phénotype luminal B sont également des tumeurs hormonosensibles, mais bénéficieront en plus de l’indication de la chimiothérapie [6]. Ils représentent aussi une indication à la thérapie ciblée par le transtuzumab (Herceptine). Les phénotypes Her2 enrichie sont des tumeurs caractérisées par une forte expression pour le Her2 et l’absence de l’expression des récepteurs hormonaux. Dans notre série, la prévalence du phénotype Her2 enrichie est de 6,7%. Ces résultats sont faibles par rapport à ceux présentés dans la littérature. Par ailleurs, Al Tamimi et al. ont montré dans leur étude une prévalence de 17,3% [12]. Les tumeurs ayant un phénotype Her2 enrichie possèdent un pronostic défavorable mais sont associés à une meilleure réactivité à la thérapie ciblée au transtuzumab (Herceptin) et à la chimiothérapie à base d’antracycline [21]. Dans notre étude, le grade histologique III était le plus fréquent dans 8,5% des cas, 7,8% des cas étaient associés à un envahissement ganglionnaire et la métastase à distance a été retrouvée dans 4,2% des cas. Plusieurs études ont montré que 90 à 96% des Her2 enrichies sont associés à une surexpression du gène HER2 [21, 22]. Bull et al. ont montré qu’une augmentation de la prévalence de Her2 enrichie était associée à une augmentation de la mutation du gène p53 et que la combinaison de la mutation HER2 et p53 augmentent le risque de récidive, ainsi que la mortalité globale par comparaison avec les patientes ayant une seule mutation ou sans mutation [23]. Le phénotype basale like ou communément appelé triple négatif définit par la négativité des récepteurs hormonaux (ER négatif et RP négatif) mais exprimant les cytokératines de haut poids moléculaires (cytokératines 5, 6, 13 et 14) [24, 25]. Les tumeurs basales sont de mauvais pronostic. Elles présentent d’une part une forte instabilité génétique et seraient sensibles à des drogues provoquant des cassures de l’ADN comme les agents alkylants, les sels de platine et d’autres parts empêchant la recombinaison homologue comme les inhibiteurs de PARP. Plusieurs études ont montré que les tumeurs de phénotype basale like englobaient la plupart des mutations liées au gène BRCA1 [25, 26]. Ils présentent une grande hétérogénéité de types histologiques, caractérisés par les carcinomes médullaires, les carcinomes métaplasiques du sein et les carcinomes adénoïdes kystiques [26]. Par ailleurs, Foulkes et al. ainsi que Rakha et al. ont montré que ces groupes hétérogènes des tumeurs étaient plus fréquents chez les jeunes femmes afro-américaines et celles d’origine hispanique [27, 28]. C’est un phénotype important ou le pronostic est le plus défavorable [17]. Dans notre série, le triple négatif était présent dans 12,9% des cas. Les types histologiques les plus fréquents sont les types histologiques rares (carcinome médullaire, carcinome métaplasique, carcinome adénoïdes kystiques, etc.), le grade histologique le plus fréquent est le grade III qui a été observé dans 17,8% des cas, l’envahissement ganglionnaire a été retrouvé dans 13,7% des cas et 13,2% des cas présentaient des métastases à distances. Ces données rejoignent ceux de la littérature. D’ailleurs, Al Tamimi et al. ont montré que la fréquence des phénotypes basales est de 9,95% [12]. Un résultat similaire été observé par Bhargava et al. 10,7% [16]. Par contre, Carey et al. ont obtenu un résultat nettement supérieur (20,1%) [9]. Tan et al. ont remarqué un pourcentage élevé de l’indice de prolifération ki67 et qui est associé à la non expression des récepteurs hormonaux et un haut grade histologique des tumeurs [29]. Les traitements ciblant les récepteurs aux œstrogènes et le Her2 ne sont pas efficaces pour traiter le cancer du sein triple négatif. Des études récentes ont révélé certaines caractéristiques moléculaires pour le phénotype triple négatif dans le cancer du sein pour avoir un meilleur aperçu dans ces voies moléculaires qui peut mener à l’identification des nouveaux biomarqueurs pour avoir plus des approches thérapeutiques pour la prévention et le traitement de cette maladie [29].

## Conclusion

Cette étude nous a permis la classification phénotypique en plusieurs groupes de patients atteints du cancer du sein et pris en charge au sein du centre Mohamed VI pour le traitement des cancers, en utilisant le profil immunohistochimique des tumeurs. Le phénotype luminal A sont des tumeurs possédant un pronostic favorable, tandis que le luminal B diffère par un pronostic péjoratif vis à vis des phénotypes Her2 enrichie. Par contre, le phénotype triple négatif possède une agressivité significativement élevée par rapport aux autres profils phénotypiques. Il est à noter que l’association entre le diagnostic histologique et immunohistochimiques peut aider à déterminer le phénotype du cancer du sein, dans le but de guider le traitement et par conséquent d’améliorer la réponse au traitement.

### Etat des connaissances actuelles sur le sujet

- La curabilité du cancer du sein est en augmentation, grâce aux programmes de dépistage et aux progrès thérapeutiques;
- Mais des défis restent à relever en rapport avec l’instabilité phénotypique des cellules cancéreuses;
- Il existe plusieurs classifications moléculaires du cancer du sein selon l’expression des récepteurs hormonaux et le récepteur Her2.

### Contribution de notre étude à la connaissance

- C’est un travail qui n’a jamais été réalisé au Maroc par la classification phénotypique récente;
- La fréquence des différents types phénotypiques du cancer du sein au sein du Centre Mohammed VI pour le traitement des cancers (l’un des deux plus grand centre pour la prise en charge et le traitement des cancers au Maroc);
- L’étude de croisement des différents phénotypes avec les données épidémiologiques, cliniques et histologiques.
